# Domestic Dogs Exposed to Orthopoxvirus in Urban Areas of Brazil

**DOI:** 10.3390/v17010131

**Published:** 2025-01-17

**Authors:** Débora de Meneses, Ana G. Stoffella-Dutra, Vicenzo S. Blaso, Iara M. de Almeida, Karolina L. Dias, Iago José da S. Domingos, Gabriela P. Ribeiro, Wendel Coura-Vital, Alexandre B. Reis, Thallyta M. Vieira, Giliane de S. Trindade

**Affiliations:** 1 Laboratório de Vírus, Departamento de Microbiologia, Instituto de Ciências Biológicas, Universidade Federal de Minas Gerais, Avenida Antônio Carlos, 6627, Belo Horizonte 31270-901, Brazil; 2 Departamento de Análises Clínicas, Escola de Farmácia, Campus Morro do Cruzeiro, Universidade Federal de Ouro Preto, Morro do Cruzeiro, s/n, Ouro Preto 35402-163, Brazil; 3 Departamento de Biologia Geral, Universidade Estadual de Montes Claros, Avenida Cula Mangabeira, Santo Expedito 39401-001, Brazil

**Keywords:** zoonosis, vaccinia virus, ecoepidemiology, companion animals, serology, zoonotic poxviruses

## Abstract

Domestic animals can share viral pathogens with humans, acting mainly as a bridge host. The *Orthopoxvirus* genus hosts important zoonotic species that have emerged in urban areas worldwide. Nevertheless, the role of companion animals, such as dogs and cats, in the circulation of orthopoxviruses in urban areas remains poorly understood. Therefore, the objective of this study was to evaluate the presence of neutralizing anti-orthopoxvirus antibodies in serum samples from owned dogs from three municipalities in Minas Gerais, as well as the presence of the C11R and A56R orthopoxviruses genes. The presence of neutralizing antibodies was detected in 14.3% of the animals investigated. However, no sample was positive for the presence of the genes investigated. Further study of the population of dogs in urban areas may prove a valuable tool for understanding the spread of orthopoxviruses in urbanized areas of Brazil.

## 1. Introduction

Zoonotic diseases are among the greatest threats to global public health [1]. More than 70% of emerging diseases originate from animals, with wildlife being the main source of these diseases. Domestic animals are the source of 25% of zoonoses [2,3,4].

Since the first human settlements, zoonoses have become more frequent after the beginning of the animal domestication process and have, in a way, shaped human evolution [5,6,7]. Several factors contribute to the importance of domestic animals in transmitting zoonotic pathogens, even livestock and companion animals. Domestic animals have close contact with humans, a wide geographical distribution, and high abundance. These characteristics of domestic animals increase the sharing of pathogens [8,9]. According to Morand, McIntyre and Baylis [10], the longer the domestication time, the greater the number of pathogens shared with humans and other domestic animals. The study by Johnson et al. [11] showed that domestic animals harbor 50% of the zoonotic viral species of mammals and harbor a median of 19.3 zoonotic viruses, while the mean for wild animals is 0.23. In addition, network analyses showed that domestic animals occupy a central position in the sharing of zoonotic pathogens from wild animals to humans [11,12,13], therefore acting as a bridge host [14,15].

Companion animals or pets are animals that humans have an emotional relationship with. These animals can help people with disabilities, perform tasks (as service animals), and treat psychological conditions (as emotional support animals) [16,17]. Dogs, belonging to the order Carnivora, are the most popular companion animals and are found in a high density in urban areas. These animals can be free-roaming or under the care of guardians/owners [18]. Most owned dogs access the outdoors without supervision [19,20]. The canine population had been growing in recent decades around the world [21,22]. According to Abinpet [23], there are 167.6 million pets in Brazil and dogs comprise 40.4% of this total.

Concerning viral zoonoses, dogs are implicated in the transmission of canine parvovirus, canine distemper virus, canine coronavirus, and canine herpesvirus to wild animals [24,25] and, notably, rabies virus to wildlife and humans, especially in urban areas [4,15]. Dias et al. [26] found dogs, horses, and cattle are being exposed to the Mayaro and Oropouche viruses in urban and peri-urban areas of the Brazilian Centro-Oeste region. Similarly, Davila et al. [27], found a high prevalence of neutralizing antibodies to West Nile virus in dogs from urban areas of Mexico. Despite this, the dogs showed no signs of the disease and there were no reports of infections in humans during the period in which they were sampled. These data show how domestic dogs can be important sentinels for human diseases in urban areas [4,15].

A group of zoonotic viruses impacting public health globally is the *Orthopoxvirus* genus. Wild and domestic animals participate in orthopoxvirus maintenance and transmission to humans in urban areas. The best-known member of this genus is the variola virus, which caused smallpox and had claimed thousands of human lives over the centuries. Zoonotic orthopoxviruses important to human and animal health are cowpox virus, monkeypox virus and vaccinia virus [28,29]. Other orthopoxviruses that have been reported in humans are the camelpox virus, Akhmeta virus, and Alaskapox virus [30,31].

The recent emergence of the monkeypox virus (species *Orthopoxvirus monkeypox*), which causes mpox, across the globe from 2022 highlights the importance of orthopoxviruses. Since the first descriptions of monkeypox virus infections in humans, the occurrence of cases has been concentrated in rural areas, close to wilderness areas. Most of these cases are associated with the zoonotic transmission of the monkeypox virus, mainly through contact with rodents, possible reservoirs, and wild primates [28,32]. As of 2017, the mpox outbreak in Nigeria, a country where there had been no recorded cases of the disease for almost 40 years, has shown important epidemiological changes that are also seen in the global outbreak. Among these changes are the fact that most human cases occur in urban areas and person-to-person transmission has become more common [32,33,34,35]. The involvement of companion animals in the transmission of the monkeypox virus lacks evidence. To date, there have been only two reports of domestic dogs that likely became infected after contact with mpox-positive owners [36,37]. Morgan et al. [38] collected skin swab or fur samples from the pets of infected owners, and four dogs and one cat tested positive for monkeypox virus and RNase-P DNA. No animals with positive monkeypox samples had a viable virus or orthopoxvirus antibodies, indicating that the animals were not infected but likely contaminated by infected humans within the household, according to the authors.

Cowpox virus (species *Orthopoxvirus cowpox*) circulates mainly in Europe, infecting cats, humans [39,40,41] and confined wild animals in urban areas [42]. The main form of transmission to humans reported is contact with domestic cats. Cats become infected by hunting and preying on rodents, which are reservoirs of the virus [39,40,41]. Cases of infection in dogs and other canids are less common, but these animals can show signs of the disease [43,44,45,46].

Vaccinia virus (species *Orthopoxvirus vaccinia*) circulates mainly in South America, with Brazil presenting the most reported cases. This virus causes bovine vaccinia in cattle and workers in rural areas [47,48,49,50]. Some recent studies have shown the silent circulation of the virus in wild animals, such as coatis [51] and capybaras [52,53], domestic dogs [51], domestic cats [54], *Rattus rattus* [55] and the human population present in urban areas of Brazil [56]. Minas Gerais is the Brazilian state that, since the emergence of vaccinia in Brazil in the late 1990s, has recorded the highest number of cases of bovine vaccinia in humans and animals [50,57,58]. The state has a significant dairy economy and artisanal cheeses of international cultural value, which could be affected by the impacts of bovine vaccinia. Despite this, Minas Gerais and Goiás are the only Brazilian states where notification of cases is mandatory [50]. Even so, the scenario of vaccinia virus circulation has many gaps.

In this context, the urban environment has a large population that has not been vaccinated against smallpox or mpox; therefore, it is susceptible to orthopoxvirus infections. As well, a portion of the urban population may be immunocompromised, which can present severe symptoms [49]. Also, the high density of pets in this environment and the greater proximity to humans highlight the need for further research into the circulation of vaccinia virus in these populations.

Therefore, the objective of this study was to retrospectively evaluate the circulation of orthopoxvirus in urban areas of Minas Gerais, Brazil, through owned dogs. To this end, we investigated the presence of neutralizing antibodies and the viral genome in serum samples from dogs living in urban areas in three municipalities of Minas Gerais.

## 2. Materials and Methods

### 2.1. Areas of Study and Companion Animal Samples

The convenience collections analyzed consisted of serum or plasma samples from 318 urban owned dogs, collected between 2008 and 2020 (Table 1). The samples of dogs included in the present study were obtained from animals living in urban areas. Blood samples were collected as described by Coura-Vital et al. [59] and Leal et al. [60]. Most individual animals were females (164/51.6%) and adults (228/71.7%). This study adopted two age categories: puppies, animals ≤12 months, and adults, >12 months.

**Figure 1:**
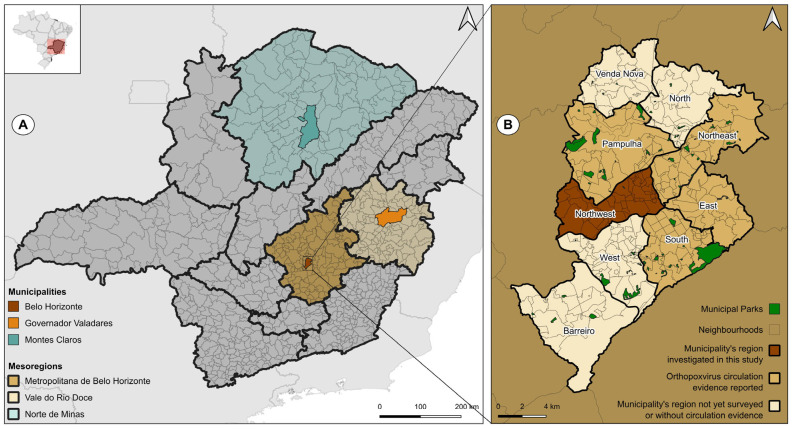
Locations of municipalities where samples were obtained from owned dogs. (**A**) The map of Minas Gerais displays the municipalities investigated and their respective mesoregions. (**B**) The map of Belo Horizonte and its regions illustrates the data available on the urban circulation of orthopoxvirus in four of the nine regions. This study presents the first investigation of the northwest region. The maps were constructed with Universal Transverse Mercator (UTM 22S-24S), DATUM SIRGAS 2000, cartographic base: [57]. Created with QGIS Software, version 3.24.3.

The animals were sampled in three municipalities of Minas Gerais: Belo Horizonte, the state capital, Montes Claros and Governador Valadares. These municipalities were investigated in our study because they are in different state mesoregions (Figure 1A and Table 2) [61]. In addition, it has been documented that orthopoxviruses are present in the urban environment of four distinct regions within the city of Belo Horizonte (Figure 1B). Differently, data concerning orthopoxvirus circulation in Montes Claros and Governador Valadares are limited. The straight-line distance between Montes Claros and Belo Horizonte is 353.93 km and it is 242.24 km between Governador Valadares and Belo Horizonte [62].

Belo Horizonte had a total population of 2,315,560 inhabitants in 2022 (Table 2) and is located in the Belo Horizonte Metropolitan mesoregion (Figure 1A) [61]. Approximately 82% of the city’s area consists of urbanized regions. The city is situated between two biomes: the Cerrado and the Atlantic Forest.

The city of Montes Claros is situated within the Norte de Minas mesoregion (Figure 1A) and registered a population of 414,240 in 2022 [61]. Only 2.04% of the total territory has been urbanized (Table 2) [61]. The city is situated between two distinct biomes, the Caatinga and the Cerrado, and thus represents a transition zone between these two ecological systems.

The city of Governador Valadares is located in the Vale do Rio Doce mesoregion (Figure 1A). The city’s population was 257,171 in 2022 (Table 2). In terms of urbanization, only 2.13% of the territory is urbanized [61]. The city is part of the Atlantic Forest biome.

### 2.2. PRNT

A total of 314 serum/plasma samples from dogs were subjected to the plaque reduction neutralization test (PRNT) to investigate the presence of neutralizing anti-orthopoxvirus antibodies, which is considered the standard technique for this purpose. The followed protocol was described by Newman et al. [63], with modifications previously published by Kroon et al. [64]. Samples from 4 of the 318 dogs did not have enough volume for serological analysis. All these animals were from Montes Claros.

BSC-40 cells were implanted into 6-well plates and maintained in Eagle’s minimum essential medium supplemented with 5% fetal bovine serum, 100 mg/mL streptomycin, 100 IU/mL penicillin, and 1 mg/mL amphotericin B. The BSC-40 cell was obtained from the Laboratório de Vírus cell bank. The serum was diluted at 1:20, tested in duplicate and incubated with vaccinia virus Western Reserve strain (VACV-WR). Samples with a volume of less than 100 µL available were tested at a dilution of 1:40. Samples considered positive were those with ≥50% reduction of lysis plates compared to the negative serum control (composed of fetal bovine serum).

### 2.3. qPCR

The orthopoxvirus genes investigated were C11R, which encodes the viral growth factor (VGF), and A56R, which encodes the viral hemagglutinin (HA) [60,61]. The primers sequences were, respectively: VGF-F 5′-CGCTACAACAGATATTCCAGCTATCAG-3′ and VGF-R 5′-AGCGTGGATACAGTCACCGTGTAA-3′; HA-gen F 5′-CATCATCTGGAATTGTCACTACTAAA-3′ and HA-gen R 5′-ACGGCCGACAATATAATTAATGC-3′. The chemical and physical conditions employed in the reactions were based on those described by Trindade et al. [65] and Kroon et al. [64], with modifications. Serum or plasma samples were diluted in a proportion of 1:10 in phosphate-buffered saline 1X and tested directly via qPCR. The qPCRs were carried out on Step One™ and QuantStudio™ 3 and 6 equipment (Applied Biosystems, Thermo Fisher Scientific, São Paulo, Brazil). 

A total of 318 samples from owned dogs were tested in duplicate and each reaction was conducted in a final volume of 10 µL. The cycling conditions for amplification of the C11R and A56R genes were 95 °C for 10 min for initial denaturation, 40 cycles of denaturation at 95 °C for 10 s, and pairing and extension at 58 °C for 40 s. To construct the denaturation curve, heating at 95 °C for 15 s, cooling at 58 °C for 15 s, and heating again at 95 °C for 15 s were performed. The detection system was SYBR^®^ Green I. Samples considered positive would meet the following criteria: amplification in both duplicates, amplification in duplicates during repetition, mean Cq < 38, and mean denaturation temperature (Tm) range ± 1 °C to the positive control [64]. The VACV-WR strain was used as a positive control.

## 3. Results

Of the 314 serum or plasma samples subjected to the PRNT, 45 samples (14.3%) were positive for the presence of neutralizing anti-orthopoxvirus antibodies (Table 3). The percentage reduction observed ranged from 50 to 88.2%, an average of 62.9%. As for gender, 60% of the seropositive animals were females. Moreover, 80% of seropositive individuals were adults. The mean age of the seropositive individuals was of 4.2 years (0.5–12 years). As the vaccinia virus was the orthopoxvirus circulating in Brazil prior to the arrival of the monkeypox virus in 2022, these results can indicate exposure to the vaccinia virus.

Belo Horizonte was the municipality with the highest number of seropositive animals, 31.7% (40/126), followed by Montes Claros with 7.9% (3/38) and Governador Valadares with 1.3% (2/150). Regarding the detection of the orthopoxvirus DNA, none of the 318 samples were positive for the presence of the C11R or A56R gene.

## 4. Discussion

Zoonotic orthopoxviruses are relevant to the global epidemiological scenario, as recently seen in the emergence of the monkeypox virus. A decrease in vaccination coverage against orthopoxviruses after the end of mass vaccination against smallpox is one factor pointing to this potential risk of the emergence of this viral group [56,66]. Also, anthropogenic factors, such as growing urbanization, increase the frequency of contact between the human population and domestic and wildlife populations [15,67,68]. The findings of our study indicate that owned dogs in urban regions of Minas Gerais, Brazil, are exposed to orthopoxviruses.

The changes in the epidemiology of the cowpox virus and monkeypox virus have some points in common. Cowpox virus infections in humans have historically been associated with direct contact with cattle, mainly in rural areas. Since 1970, no outbreaks caused by cowpox virus in cattle have been reported. The most affected and the main source of human infection in urban areas are domesticated or stray cats, as well as wild animals in zoos [28,42,69] As for the monkeypox virus, human cases have increased in urbanized areas of the African continent, as reported in the 2017 outbreak in Nigeria [32,34,35]. In addition, the majority of mpox cases worldwide have been detected in urban areas [70,71].

Given this scenario of other orthopoxviruses, evidence has been accumulating on the circulation of vaccinia virus in the Brazilian urban environment [51,52,53,54,56,72]. In 2012, an analysis of capybara feces found in a green area in Belo Horizonte detected the presence of vaccinia virus in Minas Gerais’s urbanized areas. Our findings suggest that the vaccinia virus may have been present there previously. Following this chronology, the data suggest urban circulation in other municipalities in Minas Gerais, such as Governador Valadares in 2014–2015 and Montes Claros in 2020, despite the low rates of seropositivity detected in these municipalities in our study (1.3 and 7.9%, respectively). This study marks the first time the vaccinia virus has been detected in Governador Valadares and Montes Claros, as well as a new region of Belo Horizonte, the northwest [54,72].

Furthermore, dogs and cats have been investigated in the context of vaccinia virus circulation in rural areas of Brazil [73,74,75]. Peres et al. [73,75] investigated the presence of vaccinia virus in farms with and without reported outbreaks in the state of São Paulo. They found antibodies against orthopoxvirus in 22.8% of the dogs sampled and in one cat. The presence of C11R was not detected in any of these animals. Peres et al. [74] found the presence of the A56R gene in three dogs from two farms with reported cases of bovine vaccine. Nonetheless, no clinical signs were observed in the animals. 

Anti-orthopoxvirus neutralizing antibodies were detected in 14.3% of dogs tested in our study. Costa et al. [51] found seropositivity of 19% (35/184) in owned dogs in Vila Marçola, Belo Horizonte, but a cut-off point of ≥70% was adopted. Vila Marçola is close to Mangabeiras Park, a large natural green space in the city. The authors also found coatis positive for the presence of vaccinia virus DNA and anti-orthopoxvirus neutralizing antibodies (14.4%, 13/90) within Mangabeiras Park. These findings emphasize that green spaces such as parks can be an important contact interface between owned free-roaming cats and dogs, wild species that host the virus, and humans [25,68]. Among the seropositive dogs in Vila Marçola, 20% were positive for the presence of the C11R and A56R genes in the serum. Additionally, DNA vaccinia virus was detected in an anal swab sample from one individual, which may be another route of excretion of the virus into the environment [51].

Costa et al. [54] analyzed serum samples from 277 urban owned cats from five Brazilian states, but only the animals from Belo Horizonte had evidence of exposure to vaccinia virus. The authors found a seropositivity rate of 5.8%. This finding aligns with our own, in which Belo Horizonte exhibited the highest seropositivity rate. The authors also found vaccinia virus DNA in 4.7% of the animals analyzed.

There is no robust evidence for the role of domestic animals in the circulation and maintenance of vaccinia virus in urban areas, nor for how infection occurs in these animals. Therefore, further studies should be conducted to assess whether dogs could be a source of infection for humans. Our findings strengthen the potential of dogs as sentinels for zoonotic diseases [4,15]. Dogs as sentinels have already been proposed for some flaviviruses [27,76,77], alphaviruses and orthobunyaviruses [26]. Surveillance of sentinel populations can be an important tool for incorporating the One Health concept into research. In addition, sentinels can indicate viruses spread over time and space, which could be a valuable addition to monitoring wild populations [78]. 

According to Halliday et al. [79], three features define a potential sentinel: first, the sentinel population must develop a detectable response to the pathogen, such as the production of antibodies or the detection of pathogen presence; second, an epidemiological, spatial or ecological connection between the sentinel population and target population must exist; and third, there must be a route of transmission of the pathogen to these populations. Within the context of Minas Gerais, dogs have the potential to act as sentinels for orthopoxvirus in urban settings. Domestic dogs residing in urban areas have developed detectable responses to orthopoxvirus, as evidenced by the seroconversion in our study. Moreover, these animals have proximity to humans, who may be infected with orthopoxviruses. However, the transmission route of orthopoxvirus to these animals remains unclear. It is noteworthy that our study utilized convenience samples, which have certain limitations. No data were collected that could indicate how and where these animals were exposed to orthopoxviruses in urban areas, nor the frequency of their outside access. Conversely, the human population in each of the municipalities studied indicates that more animals could be sampled and tested. Additionally, stray pets may be an appropriate population to investigate the orthopoxvirus circulation in urban settings, as they may have more opportunities for virus exposure due to roaming freely.

## Figures and Tables

**Table 1:** Collections of owned dogs from urban areas of Minas Gerais, Brazil, were analyzed in this study.

City	Mesoregion	Collection Date	Sample Type	Number of Individuals
Belo Horizonte	Metropolitana de Belo Horizonte	2008–2009	Plasma	126
Montes Claros	Norte de Minas	2020	Serum	42
Governador Valadares	Vale do Rio Doce	2014–2015	Serum	150
				Total: 318

**Table 2:** Demographic, economic, and environmental characteristics of the municipalities included in this study. Data are from the Brazilian Institute of Geography and Statistics [61].

	Municipalities		
	Belo Horizonte	Montes Claros	Governador Valadares
**Mesoregion**	Metropolitana de Belo Horizonte	Norte de Minas	Vale do Rio Doce
**Territory (km^2^)**	331,354	3,589,811	2,342,376
**Urbanized area (km^2^)**	274,04	73.51	49.93
**Biome**	Cerrado and Atlantic Forest	Caatinga and Cerrado	Atlantic Forest
**Human population**	2,315,560	414,240	257.171
**Demographic density (hab/km^2^)**	6988.18	115,39	109.79

**Table 3:** Urban owned dogs that were positive for anti-orthopoxvirus neutralizing antibodies in the serum or plasma samples.

City	Sample	Collection Date	Sex	Age (Years)	PRNT		qPCR	
					Dilution Tested	% of Plaque Reduction	C11R	A56R
Montes Claros	CA32	2020	M	3	1:40	62.8	-	-
	CA40		F	8.6	1:40	65.7	-	-
	TMV03		M	6	1:40	80.23	-	-
Governador Valadares	347	2014–2015	M	2	1:20	88.01	-	-
	360		M	3	1:20	64.2	-	-
Belo Horizonte	41	2008	F	10	1:20	68.95	-	-
	46		F	1	1:20	65.28	-	-
	70		M	2	1:20	58.5	-	-
	91		F	2	1:20	52.63	-	-
	122		F	12	1:20	63.46	-	-
	128		M	6	1:20	78.95	-	-
	130		F	1	1:20	62.32	-	-
	132		F	2	1:20	77.73	-	-
	195		M	2	1:20	72.69	-	-
	204		M	5	1:20	60.36	-	-
	208		F	2	1:20	68,38	-	-
	209		F	8	1:20	71.7	-	-
	211		M	6	1:20	52.38	-	-
	261		F	2	1:20	73.6	-	-
	264		F	NI *	1:20	54.85	-	-
	281		M	8	1:20	51.42	-	-
Belo Horizonte	282	2008	F	6	1:20	58.02	-	-
	284		M	7	1:20	66.67	-	-
	286		M	4	1:20	56.37	-	-
	302		F	4	1:20	51.93	-	-
	308		F	1	1:20	59.02	-	-
	314		M	8	1:20	66,34	-	-
	319		M	5	1:20	57.32	-	-
	320		F	3	1:20	57.22	-	-
	321		F	8	1:20	59.54	-	-
	322		F	8	1:20	56.42	-	-
	324		F	5	1:20	51.9	-	-
	334		F	7	1:20	55.58	-	-
	395		F	4	1:20	66.84	-	-
	874		M	3	1:20	59.78	-	-
	885		F	1	1:20	60.61	-	-
	945		F	6	1:20	50	-	-
	1147		F	0.8	1:20	56.58	-	-
	1429		F	2	1:40	54.17	-	-
	1436		M	0.7	1:40	50	-	-
Belo Horizonte	2063	2009	M	1	1:40	59.83	-	-
	2129		M	2	1:40	66.54	-	-
	2249		F	5	1:40	69.23	-	-
	2273		F	2	1:40	57.48	-	-
	2312		F	1	1:40	88.19	-	-
	Total: 45					Mean: 62.9		

* NI: not informed.

## Data Availability

The data presented in this study are available within the article.
